# Development and validation of a prognostic score during tuberculosis treatment

**DOI:** 10.1186/s12879-017-2309-9

**Published:** 2017-04-08

**Authors:** Eric Walter Pefura-Yone, Adamou Dodo Balkissou, Virginie Poka-Mayap, Hadja Koté Fatime-Abaicho, Patrick Thierry Enono-Edende, André Pascal Kengne

**Affiliations:** 1 Department of Internal medicine and specialities, Faculty of Medicine and Biomedical Sciences, University of Yaounde 1, Yaounde, Cameroon; 2 Yaounde Jamot Hospital, P.O Box: 4021, Yaounde, Cameroon; 3 Institut Supérieur de Technologie Médicale, Yaoundé, Cameroon; 4 Medical Research Council & University of Cape Town, Cape Town, South Africa

**Keywords:** Tuberculosis, Mortality, Prediction, Risk score

## Abstract

**Background:**

Death under care is a major challenge for tuberculosis (TB) treatment programs. We derived and validated a simple score to predict mortality during tuberculosis treatment in high endemicity areas.

**Methods:**

We used data for patients aged ≥15 years, diagnosed and treated for tuberculosis at the Yaounde Jamot Hospital between January 2012 and December 2013. Baseline characteristics associated with mortality were investigated using logistic regressions. A simple prognosis score (CABI) was constructed with regression coefficients for predictors in the final model. Internal validation used bootstrap resampling procedures. Models discrimination was assessed using c-statistics and calibration assessed via calibration plots and the Hosmer and Lemeshwow (H-L) statistics. The optimal score was based on the Youden’s index.

**Results:**

A total of 2250 patients (men 57.2%) with a mean age of 35.8 years were included; among whom 213 deaths (cumulative incidence 9.5%) were recorded. Clinical form of tuberculosis (C), age (A, years), adjusted body mass index (B, BMI, kg/m^2^) and status for HIV (Human immunodefiency virus) infection (I) were significant predictors in the final model (*p* < 0.0001) which was of the form *Death risk* = 1/(1 + *e* ^− (−1.3120 + 0.0474 ∗ *age* − 0.1866 ∗ *BMI* + 1.1637 (*if smear negative TB*) + 0.5418(*if extra* − *pulmonary TB*) + 1.3820(*if HIV*+))^). The c-statistic was 0.812 in the derivation sample and 0.808 after correction for optimism. The calibration was good [H-Lχ^2^ = 6.44 (*p* = 0.60)]. The optimal absolute risk threshold was 4.8%, corresponding to a sensitivity of 81% and specificity of 67%.

**Conclusions:**

The preliminary promising findings from this study require confirmation through independent external validation studies. If confirmed, the model derived could facilitate the stratification of TB patients for mortality risk and implementation of additional monitoring and management measures in vulnerable patients.

**Electronic supplementary material:**

The online version of this article (doi:10.1186/s12879-017-2309-9) contains supplementary material, which is available to authorized users.

## Background

Tuberculosis (TB) is a major cause of death worldwide [1]. Estimates from the World Health Organisation (WHO) suggest that about 16% of the 9 million individuals who developed a TB in 2013 died of the disease, with 95% of these deaths occurring in low- and middle-income countries [2]. Determinants of death during treatment for TB have been largely characterised as recently reviewed by Wait and co-workers [3]. These determinants in developing countries with high endemicity for TB and high prevalence of HIV infection comprise essentially the co-infection TB/HIV, severe HIV related immune-depression, smear negative pulmonary tuberculosis (PTB−), under-nutrition and low socio-economic status [3]. In developed countries, determinants of mortality during TB treatment are mostly non-infectious comorbidities, smear positive pulmonary TB (PTB+), alcohol and drugs abuse [3].

In 2013, Horita and co-workers derived a risk score to predict mortality in Japanese aged on average 65 years, receiving in-hospital care for smear positive pulmonary tuberculosis [4]. However, their cohort did not include patients with HIV infection, and did not account for other clinical forms of tuberculosis (PTB− and extra-pulmonary TB). Furthermore, we are not aware of a prognostic score integrating all major clinical forms of tuberculosis, and that has used routinely collected variables in national TB programs. Healthcare practitioners need simple and accurate tools to identify at the start of treatment, those patients with TB who are at high risk of death during treatment, and whose outcome can be modified through tailored monitoring and management. Accordingly, the aim of the present study was to develop and validate a simple score for predicting the risk of death during follow-up in patients starting TB treatment in areas of high endemicity for TB, and high prevalence of HIV infection.

## Methods

### Setting and participants

The study was conducted at the Yaounde Jamot Hospital (YJH), which is a reference hospital for TB and respiratory diseases for the Capital City of Cameroon (Yaounde) and surrounding areas. YJH hosts a diagnosis and treatment centre for TB (DTC), and an approved treatment centre for HIV infection (ATC). Over the last 5 years, the DTC of YJH has screened and treated on average 1400 to 1800 patients each year. All patients with TB aged ≥ 15 years treated with WHO categories I or II regimens between January 2012 and December 2013 were retrospectively included in this cohort study. Patients with missing data on key variables (clinical form of TB, status for HIV infection, body weight) were excluded. The study was approved by the institutional ethic review committee of the Faculty of Medicine and Pharmaceutical Sciences of the Douala University and the administrative authorities of the Health Delegation for the Centre Region.

### Classification of TB cases

Diagnosis and classification of TB at the YJH are based on the WHO’s recommendations and those of the Cameroon National TB Control Program (NTCP) [5, 6]. The following international definitions are applied at the DTC: smear-positive pulmonary tuberculosis (PTB+)–acid-fast bacilli (AFB) present in at least two sputum specimens; 2) smear-negative pulmonary tuberculosis (PTB−)–persisting negativity on two sputum examinations after a 10-day course of nonspecific antibiotic treatment in a patient with clinical and radiological signs of tuberculosis, in the absence of any obvious cause and the decision of physician to treat the patient with the full course of anti-tuberculosis drugs; 3) extra-pulmonary tuberculosis (EPTB) – tuberculosis infection involving organs other than the lungs. Patients previously treated with anti-tuberculosis medications are classified as retreatment cases and are further sub-classified as “relapse” (i.e. reoccurrence of the disease after a successful anti-tuberculosis treatment course), “failure”(i.e. positive smear after 5 months of anti-tuberculosis treatment) and “treatment after default” (i.e. starting anti-tuberculosis treatment again after two consecutive months of interruption). A “new case” refers to a patient with tuberculosis who has never received anti-tuberculosis treatment for more than 1 month previously. “Other cases of tuberculosis” refer to patients not falling in one of the categories described above. In this centre, all retreatment cases have been screened for multidrug resistant tuberculosis by Gene Xpert according to NTCP guidelines.

### Tuberculosis treatment and outcomes

Treatment of patients with active tuberculosis is based on the NTCP recommendations [5]. Patients are either hospitalised or treated on an ambulatory basis during the intensive phase of the treatment. New cases are treated for 6 months with category I regimens which comprise a 2-month intensive phase with four anti-tuberculosis [rifampicin (R), isoniazid (H), ethambutol (E) and pyrazinamide (Z)] and a 4-month continuation phase combining rifampicin with isoniazid (2RHEZ/4RH). Patients undergoing retreatment receive streptomycin (S) in addition under the category II regimen. Their duration of treatment is 8 months and comprises 2 months of REHZS, 1 month of RHEZ and 5 months of RHE (2RHEZS/1RHEZ/5RHE). All patients with tuberculosis are screened for HIV infection after informed consent has been obtained from the patient or relative for dependent patients. HIV-positive patients are prescribed prophylaxis with co-trimoxazole and combined active antiretroviral therapy (cART) free of charge. Initial antiretroviral regimens are the combinations lamivudine-zidovudine-efavirenz or lamivudine-tenofovir-efavirenz. Antiretroviral therapy was started within 2 weeks following HIV diagnosis.

At treatment completion, patients are ranked into the following mutually exclusive categories [5, 6]: 1) cured–patient with negative smear at the last month of treatment and at least one of the preceding months; 2) treatment completed–patient who has completed the treatment and for whom the smear results at the end of the last month are not available; 3) failure–patient with positive smear at the 5th month or later during treatment; 4) death–death from any cause during treatment; 5) defaulter–patient who’s treatment has been interrupted for at least two consecutive months; 6) transfer–patient transferred to complete his treatment in another center and who’s treatment outcome is unknown. Cured and treatment completed were considered as successful treatment.

### Data collection

Anti-tuberculosis treatment registers and patients charts were used for data collection. The following details were extracted: 1) demographic data including age and sex; 2) clinical data including hospitalisation during intensive phase, clinical form of TB (PTB+, PTB−, EPTB), type of patient (new case, retreatment and other), status for HIV infection (positive or negative), CD4 count in HIV positive patients; 3) anthropometric data comprising weight and adjusted body mass index (BMI) which was estimated as measured weight divided by the squared of the average sex-specific height of adult population (1.70 m in men and 1.60 m in women); and 4) the outcome of patients as treatment success, death, failure, defaulter and transfer.

## Statistical analysis

Data analysis used the R statistical software V.3.1.1 (10-07-2014) (The R Foundation for Statistical Computing, Vienna, Austria) and ‘*rms*’ and ‘*pROC*’ packages. Data are summarised as count and percentages for categorical variables and mean (or median) and standard deviation (or 25th–75th percentiles) for continuous variables. Groups’ comparisons used chi square test for categorical variables and Student’s t-test and Mann-Whitney U test for continuous variables. Logistic regressions models were used to derived the prediction models and were implemented with the *lrm* function, which fits binary logistic regression models using maximum likelihood estimation. Candidate variables were first tested for inclusion in univariable models, then the significant ones based on a *p*-value < 0.20 were all entered into the same multivariable models, and backward stepwise eliminations procedures used to retain the variables in the final models as recommended by Collett [7]. The linearity of the effects of continuous predictors was examined using restricted cubic splines. The performance of the derived model was tested on the development sample and in bootstrap resampling, which was based on 2000 replications. In the bootstrap internal validation based on 2000 replications, the original dataset was resampled at random with replacement 2000 times, with each random sample being of the same size (number of participants) as the original dataset. For each sample, the logistic regressions were fitted, the new beta coefficients estimated and a new equation derived using predictors in the final model. This new model was then tested on the dataset from which it was developed and as well as on the original dataset, and the differences in the performance on the derivation and original samples, were averaged across the 2000 replication to derive the optimism. Model’s discrimination was assessed by calculating the c-statistic in the derivation sample, and after correction for optimism in bootstrap internal validation. Model’s calibration was assessed graphically through calibration plots, and by computing the Hosmer and Lemeshow statistics (based on n-2 degrees of freedom) [8].

To facilitate the uptake of the model in clinical setting, a graphical calculator (nomogram) and a risk score table were constructed by summing up the weighted point-score from each predictor to arrive at a total score which can then be used to determine if the patient is at high risk. For this purpose, receiver-operating characteristic (ROC) curves were used to derive the optimal cut-off point score, applying the Youden’s index method [9]. Performance measures including the sensitivity, specificity, positive and negative predictive values where then estimated at this optimal threshold as well as other selected thresholds.

## Results

### Data available

Of the 3369 patients treated for TB during the study period, 384 (11.4%) had missing data on weight and/or status for HIV, the outcome was unknown in 859 (25.5%) patients including 567 who were lost to follow-up, 244 who were transferred to other centres and 48 failed on treatment; they were all excluded. Therefore, the final analytic sample comprised 2250 patients. The comparative characteristics of the included and excluded participants are summarised in Table 1. The mean age and distribution of type of patients were similar (both p ≥ 0.129), but those excluded were more likely to be male (*p* < 0.001) and to have smear negative tuberculosis (*p* = 0.008).

**Table 1:** Study participant characteristics

Characteristics	Included patients *N* = 2250 (%)	Excluded patients *N* = 1119 (%)	p
Gender			
Male	1286 (57.2)	742 (66.3)	<0.001
Female	964 (42.8)	377 (33.7)	
Age, mean (SD)	35.8 (12.7)	36.7 (13.4)	0.144
Initial hospitalization			
Yes	1363 (60.6)	495 (45.3)	<0.001
No	887 39.4)	597 (54.7)	
Clinical form			
PTB+	1527 (67.9)	724 (64.9)	0.008
PTB−	210 (9.3)	143 (12.8)	
ETB	513 (22.8)	248 (22.2)	
Type of patient			
New cases	2119 (94.2)	1037 (92.7)	0.129
Retreatment	106 (4.7)	71 (6.3)	
Other	25 (1.1)	11 (1.0)	

*SD* standard deviation, *PTB+* smear positive pulmonary tuberculosis, *PTB-* smear negative pulmonary tuberculosis, *ETB* extra-pulmonary tuberculosis

### General characteristics of included participants

The general characteristics of the included participants are shown in Table 2. Of the 2250 participants included, 1286 (57.2%) were men, and the mean age (standard deviation, SD) was 35.8 (12.7) years. Two-third of patients were PTB+ while 22.8% had extra-pulmonary TB. In all 94.2% of the participants were new cases, while 35% had HIV infection. For 352 HIV positive patients with CD4 count data available, the median (25th–75th percentiles) CD4 count was 143 (70–249.75) per mm^3^.

**Table 2:** Univariable logistic regression analysis of potential predictors of tuberculosis death

Predictors	Overall *N* = 2250	Dead patients *N* = 213 (%)	Treatment success *N* = 2037 (%)	Crude OR (95% CI)	*p*-value
Gender					0.914
Male	1286 (57.2)	121 (56.8)	1165 (57.2)	0.94 (0.74–1.31)	
Female	964 (42.8)	92 (43.2)	872 (42.8)	1	
Age, years	35.8 (12.7)	42.4 (13.5)	35.1 (12.4)	1.04 (1.03–1.05)	<0.0001
Adjusted BMI, kg/m^2^	21.4 (4.0)	19.2 (4.9)	21.6 (3.9)	0.83 (0.80–0.87)	<0.0001
Initial hospitalization					
Yes	1363 (60.6)	146 (68.5)	1217 (59.7)	1.47 (1.08–1.99)	0.012
No	887 (39.4)	67 (31.7)	820 (40.3)	1	
Clinical form					<0.0001
PTB+	1527 (67.9)	107 (50.2)	1420 (69.7)	1	
PTB−	210 (9.3)	42 (19.7)	168 (8.2)	3.32 (2.24–4.91)	
ETB	513 (22.8)	64 (30.0)	449 (22.0)	1.89 (1.36–2.62)	
Type of patient					0.393
New cases	2119 (94.2)	199 (93.4)	1920 (94.3)	1	
Retreatment	106 (4.7)	13 (6.1)	93 (4.6)	1.35 (0.74–2.4)	
Other	25 (1.1)	1 (0.5)	24 (1.2)	0.40 (0.05–2.99)	
HIV serology					
Positive	788 (35.0)	149 (69.9)	639 (31.4)	5.09 (3.74–6.93)	<0.0001
Negative	1462 (65.0)	64 (30.0)	1398 (68.6)	1	

*OR* odds ratio, *BMI * body mass index adjusted by divided weight by the square of 1.70m  for men and 1.60m for women, *SD* standard deviation, *PTB+* smear positive pulmonary tuberculosis, *PTB-* smear negative pulmonary tuberculosis, *ETB* extra-pulmonary tuberculosis

### Determinants of death

Univariable associations of baseline characteristics with death in treatment are shown in Table 2. A total of 213 deaths (cumulative incidence 9.5%) was recorded during follow-up. Furthermore, 62.9% of deaths occurred in the early phase of tuberculosis treatment (<2 months), 29.6% in the late phase, while in 7.5% of cases, the phase of death was missing. Age [OR 1.04 (95% CI: 1.03–1.05) per year higher], initial hospitalisation [1.47 (1.08–1.99)], extra-pulmonary TB [1.89 (1.36–2.62)], smear negative pulmonary TB [3.32 (2.24–4.91)], HIV infection [5.09 (3.74–6.93)] and adjusted BMI [0.83 (0.80–0.87) per kg/m^2^ higher] were associated with mortality in univariable analysis; while gender (*p* = 0.914) and type of patients (*p* = 0.393) were not. In mutually adjusted multivariable analysis, age, BMI, HIV infection and clinical form of tuberculosis (all *p* < 0.003) remained significantly associated with mortality and were retained in the final model. Restricted cubic splines analyses suggested a departure from linearity of the effects of age and BMI in both univariable and multivariable analyses. This departure was very modest for age, while the Wald test (χ^2^, *p*-value) was always in favour of very strong linear effects relative to the nonlinear effect. The χ^2^ (*p*-value) for the Wald test (linear vs. nonlinear terms) was 55.2 (<0.0001) versus 8.5 (0.037) for age, and 104.3 (<0.0001) versus 22.7 (<0.0001) for BMI in univariable analyses. Equivalents figures in multivariable analyses were 68.2 (<0.0001) versus 7.8 (0.049) for age, and 89.1 (<0.0001) vs. 20.1 (<0.0001) for BMI. In the interest of deriving a simple model for application in other settings, and to minimise the risk of overfitting from modelling complex terms of age and BMI, the linearity of the effect was assumed for age and BMI in the final model. The beta coefficients for this final model are shown in Table 3. Estimation of the risk of death based on this final model is provided by the following equation: *Death risk* = 1/(1 + *e* ^− (−1.3120 + 0.0474 ∗ *age* − 0.1866 ∗ *BMI* + 1.1637 (*if smear negative TB*) + 0.5418(*if extra* − *pulmonary TB*) + 1.3820(*if HIV*+))^).

**Table 3:** Risk factors of tuberculosis death in multivariable logistic regression model in the derivation data set (based on 213 deaths and 2037 treatment success)

Predictors	Final model		
	β-coefficient	Standard error	*p*-value
Intercept	−1.3120	0.5220	0.0120
Age, per 1 year increase	0.0474	0.0061	<0.0001
Adjusted BMI, per 1 kg/m^2^ increase	−0.1866	0.0235	<0.0001
Clinical form			<0.0001
PTB+	0		
PTB−	1.1637	0.2202	<0.0001
ETB	0.5418	0.1823	0.0030
HIV serology			
Positive	1.3820	0.1677	<0.0001
Negative	0		

*OR* odds ratio, *CI* confidence interval, *BMI* body mass index adjusted by divided weight by the square of 1.70m for men and 1.60m for women, *﻿PTB+﻿* smear positive pulmonary tuberculosis, *PTB−* smear negative pulmonary tuberculosis, *ETB* extra-pulmonary tuberculosis

### Performance of the final model

**Fig. 1:**
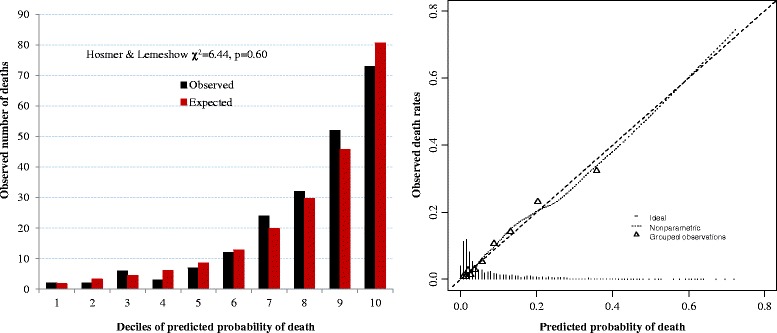
Calibration figures of tuberculosis prognostic score

**Fig. 2:**
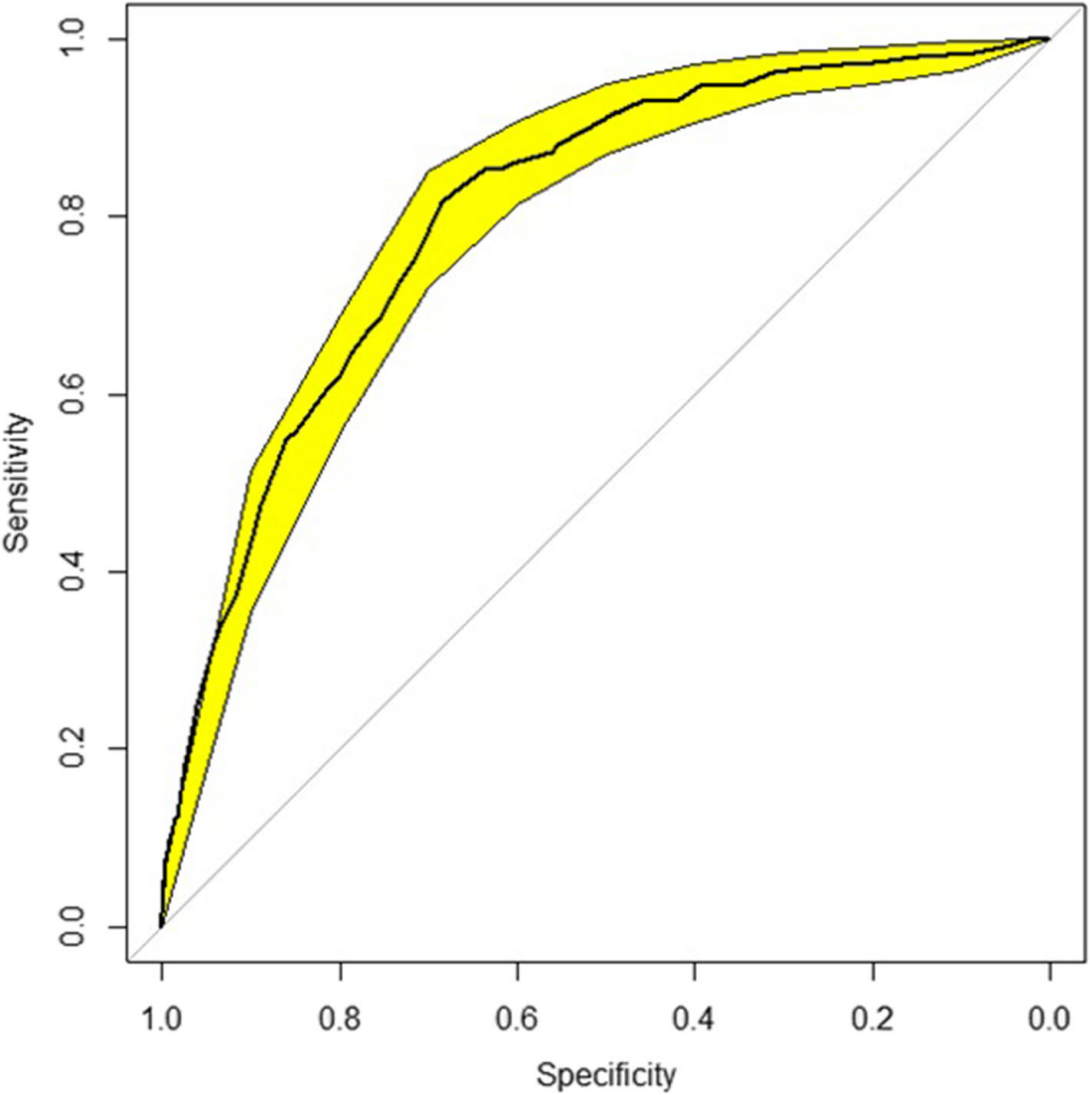
Discrimination curves of tuberculosis prognostic score

There was a good agreement between risk of death estimated by the final model and the observed rates of death, across the continuum of predicted probability (Fig. 1). The Hosmer and Lemeshow χ^2^ was 6.44 and the accompanying *p*-value was 0.60. The discrimination of the model in the derivation sample (apparent discrimination) was good with a c-statistic of 0.812 (95% CI: 0.784–0.841), with only little variability in bootstrap internal validation (Fig. 2). The optimism from 2000 samples was 0.0077 corresponding to an optimism-corrected c-statistic of 0.808. The apparent c-statistic was 0.823 when restricted cubic splines were applied to both age and BMI in the final multivariable model.

### Point scoring system

**Fig. 3:**
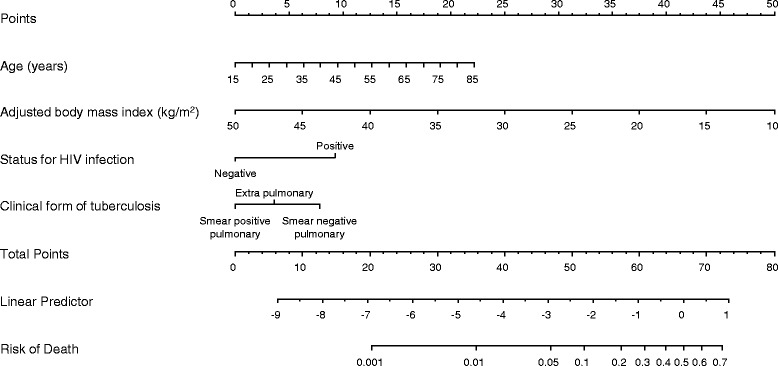
Clinical nomogram for patients starting treatment for tuberculosis, estimating the probability of dying on treatment. Instruction for using the nomogram: Locate the patient’s *age* on the *Age axis*. Draw a straight vertical line up to the *Points axis* to determine how many points the age of the patient contribute towards the predicted probability of death during TB treatment. Repeat this process for *BMI*, *status for HIV infection* and *clinical forms of tuberculosis*. Sum the points from each of the predictor to get the total points. Locate the patient’s total points on the *Total points axis*. Draw a straight line down to the *Risk of death axis* to determine the probability of dying during treatment for that patient. The risk of death is provided as a proportion and should be multiplied by 100 to obtain the value in percentage

The nomogram showing the point scores corresponding to levels of each predictor in the final model is shown in Fig. 3. The ranged of point scores was 0–22 for age, 0–50 for BMI, 0–9 for HIV infection and 0–8 for clinical forms of tuberculosis. The total points ranged from 0 to 80. The optimal point-score for predicting death was 46.5. Corresponding performance measures were 80.7% (sensitivity), 68.2% (specificity), 21.0% (positive predictive value) and 97.1% (negative predictive value). The performance of the point-scoring system at different thresholds is summarized in Table 4. The overall performance of the point-scoring system based on the c-statistic was 0.805 (0.775–0.834). The risk of death in the form of probability can be obtained from the point-score via the following formula *Death risk* = 1/(1 + *e* ^− ((*total points* − 66.5) ∗ 0.1493168)^), where 0.1493168 is the linear predictor unit per point; and 66.5 the scaling factor for generating only positive point scores. The total points score can be obtained from linear score by using the formula *total points* = 6.6971700 ∗ *linear predictor score* + 66.5. Additional file 1: Table S1 shows how to estimate the risk of deaths from the point-scoring system.

**Table 4:** Performance at different score thresholds of tuberculosis prognostic point scoring

Threshold	Risk of death	sensitivity	specificity	Positive predictive value	Negative predictive value
46.5 (optimal)	4.8%	80.7	68.2	21.0	97.1
25	0.2%	100	0.6	9.5	100
30	0.4%	99.5	3.7	9.7	98.7
35	0.9%	97.6	20.7	11.4	98.8
40	1.9%	93.4	37.4	13.5	98.2
45	3.9%	85.6	61.5	18.9	97.7
50	7.8%	68.1	76.6	23.3	95.8
55	15.2%	41.8	90.3	31.0	93.7
60	29.0%	17.4	97.5	42.5	91.9
65	44.3%	5.2	99.7	64.7	90.9
70	62.7%	0.9	100	100	90.6

## Evaluation of the risk of death

Based on the optimal point-score of 46.5 in the derivation sample, the four levels of risk described in Table 5 were established. Class I corresponds to low risk and class IV to critical risk. As expected death rate increased across risk classes, being 1.6, 6.8, 15 and 36.5% respectively for Class I, II, III and IV.

**Table 5:** Discriminative performances of tuberculosis simple prognostic score (CABI) and risk categories

Parameters	Values
Linear predictor score (CABI), mean (standard deviation)	−2.89 (1.31)
C-statistic by linear predictor score (CABI) (95% CI)	0.812 (0.784–0.841)
C-statistic by risk group	0.788 (0.758–0.818)
Mortality in each risk group	
Class I, low risk, Total points < 46	16/1029 (1.6%)
Class II, moderate risk, 46 ≤ Total points < 50	25/366 (6.8%)
Class III, high risk , 50 ≤ Total points < 60	106/674 (15%)
Class IV, critical risk , Total points ≥ 60	66/181 (36.5%)

CABI, derived from clinical form of tuberculosis (C), Age (A), Body mass index (B), I (HIV infection)

*Death risk* = 1/(1 + *e* ^− (−1.3120 + 0.0474 ∗ *age* − 0.1866 ∗ *BMI* + 1.1637(*if smear negative TB*) + 0.5418(*if extra* − *pulmonary TB*) + 1.3820(*if HIV*+))^)

*Total points* = 6.6971700 ∗ *linear predictor score* + 66.5

## Discussion

We have derived a model to predict fatal outcomes in patients starting treatment for tuberculosis in settings of high endemicity for tuberculosis and HIV infection. The derived model used easily assessable variables in routine clinical practice. These predictors were age, clinical form of tuberculosis, body mass index and status for HIV infection. The model developed and the derived point-scoring system with these predictors had near perfect calibration and good discrimination, with only marginal variation in bootstrap internal validation.

Predictors retained in our final model were essentially those previously known to be associated with mortality risk in tuberculosis [10–21]. In both developed and developing countries, advancing age for instance has been widely reported to be associated with higher mortality risk in patients with tuberculosis. Possible reasons for this association include late diagnosis of TB at the advanced stage of the disease in older people, unfavourable living conditions, and comorbidities. Lower BMI is a potent marker of under-nutrition, a well-known determinant of mortality risk in patients with tuberculosis. Various markers of under-nutrition have been associated with excess mortality in patients with TB, including low BMI or body weight and hypoalbuminemia [4], and a composite score of under-nutrition comprising low BMI, hypoalbuminemia, hypocholesterolemia, and lymphopenia [22].

Available studies have also linked extra-pulmonary TB with higher risk of mortality [17], with the magnitude of such risk being two to five times higher when compared with mortality from smear positive TB. Likewise, smear negative TB is associated with two to three times higher mortality rates, relative to smear positive TB [17]. Co-infection with HIV has also been established as a powerful determinant of death in patients with TB, with the magnitude ranging from two to nearly eight folds when compared to HIV-free patients [13, 14, 17, 23–27].

We did not test the radiographic predictors. It should however be recalled that radiographic features of TB tend to be correlated with indicators of TB severity such as low BMI [28, 29]. Therefore, loss of discriminatory information subsequent to not including radiographic predictors in our model, if any, is likely marginal.

The final model developed in the current study was based on four predictors that are routinely assessed in most tuberculosis control programs in high endemicity areas. Interestingly, data on those predictors can be accurately collected event by lay people, meaning that the model will be applicable even in remote and underequipped primary care settings. This model had excellent calibration and good discrimination both in the derivation sample and in bootstrap internal validation. The observed discriminatory power of the model (AUC ≥ 0.80) suggest that any drop in this performance property of the model when applied to other settings, will need to be sizable to push the model below the unacceptable zone (AUC < 0.70); which is very unlikely. However, due to the anticipated difference in death incidence across settings, it is an expectation that the model may require some adjustment where appropriate in order to maintain optimal calibration properties [30]. There are practical examples to support that this adjustment is easily achieved through simple intercept recalibration [31].

Our receiver operating characteristic curves analysis identified the absolute risk threshold of around 5% as estimated by the derived model, to be the optimal threshold to identify at the start of treatments, those patients with TB who are at increased risk of death during follow-up. In TB patients scoring above this threshold, active investigation and timely management of modifiable co-factors know to be associated with mortality risk has a potential to improve survival among patients starting TB treatment. Such co-factors include and are not restricted to under-nutrition, anaemia and opportunistic diseases. Considering that TB tend to predominantly affect socially deprived people [32, 33], early nutritional support should be an integral component of TB treatment programs. Similarly, early introduction of antiretroviral therapy has been shown to reduce the risk of death by about 68% in TB patients co-infected with HIV [34].

A sizable proportion of potentially eligible participants had missing data on the outcome of candidate predictors of interest and was excluded. While data imputation technics could be used to fill the missingness and possibly using data from all eligible participants, implementing some steps of predictive models development on imputed data is not straightforward [35]. However, the similarity of profiles between excluded participants and those in the final analytic sample suggest that the derived estimates are likely unbiased. By using bootstrap resampling for internal validation, we were able to use all participants with valid data for model development, therefore achieving a very high number of outcomes per candidate variable tested, and accordingly a very good statistical power [35]. In the absence of individual level data on height, the adjusted BMI was used as predictor in our model. By constraining the height to assume the same gender-specific value in all participants, we have possibly reduced to some extent the variability in the true BMI across participants, which in turn could lead to non-optimal extraction of predictive information from BMI. However the overall good discriminatory power achieved using this imperfect BMI suggests that further gain from using the true BMI will likely be marginal. The non-appreciable difference in discriminatory powers between our final model and a model accounting for possible non-linearity of the effect of age and BMI through restricted cubic splines, confirms that essential discriminatory information from the two predictors are captured by the linear forms. This supports our choice of a simplified final model assuming a linear functional form of the two predictors.

## Conclusions

We have developed a simple risk score based on four routinely collected variables in TB program, to estimate the chance of death in patients commencing TB treatment in high endemicity areas for TB and HIV infection. This model had good performance in the derivation sample and during internal validation using bootstrap resampling methods. Successful application of this model in routine TB care settings can assist the identification of a group of patients whose risk of fatal outcome can potentially be modify through identification and correction of co-factors of mortality in TB. To promote the uptake of the model in routine setting, we have developed a graphical calculator in the form of a nomogram as well as a point scoring system to allow risk estimation of the model without manipulating complex formula. The model is hereafter called CABI, reflecting the four variables in the models: C for ‘clinical form of tuberculosis’, A for ‘age’, B for ‘body mass index’ and I for ‘status for HIV infection’. Just like any newly developed model, results from this study are just preliminary findings. CABI will require independent external validation to establish the performance both in the study setting and in other settings including African, Latin America and Asian populations [36], before recommendation for uptake in routine clinical setting.

## Additional file

Additional file 1: Table S1. Point scoring for a death prediction score in tuberculosis patients. (DOCX 19 kb)

## Abbreviations

BMI: Body mass index
cART: combined active antiretroviral therapy
DTC: Diagnosis and treatment centre for TB
E: Ethambutol
EPTB: Extra-pulmonary tuberculosis
H: Isoniazid
HIV: Human immunodefiency virus
H-L: Hosmer and Lemeshwow
NTCP: Cameroon National TB Control Program
PTB−: Smear negative pulmonary tuberculosis
PTB+: Smear positive pulmonary TB
R: Rifampicin
ROC: Receiver-operating characteristic
TB: Tuberculosis
WHO: World Health Organisation
YJH: Yaounde Jamot Hospital
Z: Pyrazinamide
