# Supplementary material for: Development and validation of a prognostic score during tuberculosis treatment
Source: BMC Infect Dis. 2017 Apr 8;17:251. doi: 10.1186/s12879-017-2309-9 (PMC5385091; doi:10.1186/s12879-017-2309-9)
Supplement: Additional file 1: Table S1. — Point scoring for a death prediction score in tuberculosis patients. (DOCX 19 kb) [file 12879_2017_2309_MOESM1_ESM.docx]

**Table S1: Point scoring for a death prediction score in tuberculosis patients**

| **Age (years)** | |  | **Adjusted BMI (kg/m^2^)** | |  | To convert the point score into risk of death, look at the correspondence below | |
| --- | --- | --- | --- | --- | --- | --- | --- |
| Range | points |  | **Range** | **Points** |  |  |  |
| <20 | 0 |  | <=10 | 50 |  |  |  |
| [20-25[ | 2 |  | ]10-15] | 44 |  | **Total points** | **Risk of death** |
| [25-30[ | 3 |  | ]15-20] | 38 |  | <=20 | <=0.1% |
| [30-35[ | 5 |  | ]20-25] | 31 |  | 36 | 1% |
| [35-40[ | 6 |  | ]25-30] | 25 |  | 40 | 2% |
| [40-45[ | 8 |  | ]30-35] | 19 |  | 43 | 3% |
| [45-50[ | 10 |  | ]35-40] | 12 |  | 45 | 4% |
| [50-55[ | 11 |  | ]40-45] | 6 |  | 47 | 5% |
| [55-60[ | 13 |  | >45 | 0 |  | 52 | 10% |
| [60-65[ | 14 |  |  |  |  | 55 | 15% |
| [65-70[ | 16 |  | **Clinical forms of TB** | |  | 57 | 20% |
| [70-75[ | 17 |  | **Forms** | **Points** |  | 59 | 25% |
| [75-80[ | 19 |  | PTB+ | 0 |  | 61 | 30% |
| [80-85[ | 21 |  | ETB | 4 |  | 62 | 35% |
| 85+ | 22 |  | PTB- | 8 |  | 64 | 60% |
|  |  |  |  |  |  | 65 | 45% |
|  |  |  | Sum up the points across the all 4 factors above to obtain the total point score | |  | 67 | 50% |
| **HIV status** | |  |  |  |  | 68 | 55% |
| **Status** | **Points** |  |  |  |  | 69 | 60% |
| Negative | 0 |  |  |  |  | 71 | 65% |
| Positive | 9 |  |  |  |  | 72+ | >=70% |

*BMI* body mass index adjusted by divided weight by the square of 1.70m for men and 1.60m for women, *TB* tuberculosis, *PTB+* smear positive pulmonary tuberculosis, *PTB−* smear negative pulmonary tuberculosis, *ETB* extra-pulmonary tuberculosis
